# Interaction of the periplasmic chaperone SurA with the inner membrane protein secretion (SEC) machinery

**DOI:** 10.1042/BCJ20220480

**Published:** 2023-02-27

**Authors:** Lucy Troman, Sara Alvira, Bertram Daum, Vicki A. M. Gold, Ian Collinson

**Affiliations:** 1 School of Biochemistry, University of Bristol, Bristol BS8 1TD, U.K.; 2 Living Systems Institute, University of Exeter, Exeter, U.K.; 3 College of Life and Environmental Sciences, Geoffrey Pope, University of Exeter, Exeter, U.K.

**Keywords:** bacterial envelope, biogenesis, chaperones, protein secretion, Sec translocon, SurA

## Abstract

Gram-negative bacteria are surrounded by two protein-rich membranes with a peptidoglycan layer sandwiched between them. Together they form the envelope (or cell wall), crucial for energy production, lipid biosynthesis, structural integrity, and for protection against physical and chemical environmental challenges. To achieve envelope biogenesis, periplasmic and outer-membrane proteins (OMPs) must be transported from the cytosol and through the inner-membrane, via the ubiquitous SecYEG protein–channel. Emergent proteins either fold in the periplasm or cross the peptidoglycan (PG) layer towards the outer-membrane for insertion through the β-barrel assembly machinery (BAM). Trafficking of hydrophobic proteins through the periplasm is particularly treacherous given the high protein density and the absence of energy (ATP or chemiosmotic potential). Numerous molecular chaperones assist in the prevention and recovery from aggregation, and of these SurA is known to interact with BAM, facilitating delivery to the outer-membrane. However, it is unclear how proteins emerging from the Sec-machinery are received and protected from aggregation and proteolysis prior to an interaction with SurA. Through biochemical analysis and electron microscopy we demonstrate the binding capabilities of the unoccupied and substrate-engaged SurA to the inner-membrane translocation machinery complex of SecYEG–SecDF–YidC — *aka* the holo-translocon (HTL). Supported by AlphaFold predictions, we suggest a role for periplasmic domains of SecDF in chaperone recruitment to the protein translocation exit site in SecYEG. We propose that this immediate interaction with the enlisted chaperone helps to prevent aggregation and degradation of nascent envelope proteins, facilitating their safe passage to the periplasm and outer-membrane.

## Introduction

Outer membrane biogenesis in Gram-negative bacteria requires targeting of the outer membrane proteins (OMPs) to the ubiquitous Sec machinery of the inner-membrane, guided by a cleavable N-terminal signal-sequence [1]. The trans-membrane proton-motive force (PMF) and ATP hydrolysis by SecA subsequently drive translocation through the hourglass-shaped SecYEG channel into the periplasm [2–4], reviewed by [5,6]. OMPs must then traverse the periplasm and peptidoglycan layer toward the β-barrel assembly machinery (BAM) for folding and insertion into the outer membrane [7,8], reviewed by [9].

Similar to the trafficking process to and across the inner-membrane, OMPs must remain unfolded during subsequent passage through the periplasm until they reach the outer-membrane. During their journey through this crowded environment [10,11], periplasmic chaperones such as SurA, Skp, PpiD and the protease DegP are recruited to help prevent and resolve aggregation [12]. Unlike cytosolic quality control factors, they must somehow operate in the absence of ATP, perhaps by virtue of their structural plasticity [13–16]. SurA is thought to be the dominant chaperone for outer-membrane delivery due to its known affinity for both the hydrophobic motifs characteristic of OMPs, and the BAM complex in the outer membrane [12,17]. Critically, the activity of SurA in inter-membrane transport depends on an interaction prior to aggregation, as unlike the Skp, it lacks the ability to recover aggregated substrates [18]. Thus, it is almost self-evident that SurA needs to interact with OMPs, prior to their release from the Sec machinery, to minimise the potential for aggregation in the periplasm. A kinetic analysis of the maturation and folding of the OMP LamB showed that *surA* mutants delay signal sequence cleavage; consistent with an interaction during, or very shortly after, translocation through the inner membrane [19].

Here, we investigate whether or not SurA makes any interaction with the inner-membrane translocation machinery, potentially for association of nascent OMPs. The core-translocon SecYEG does not have periplasmic domains large enough to accommodate SurA; however, the ancillary factors, SecDF and YidC, which associate to form the holo-translocon (HTL) do [9,20,21]. The work described here implicates these periplasmic domains in the recruitment of SurA to the translocation exit channel to help streamline the onward passage of proteins through the envelope.

## Results

### The SurA chaperone interacts with the bacterial translocon

**Figure 1.**
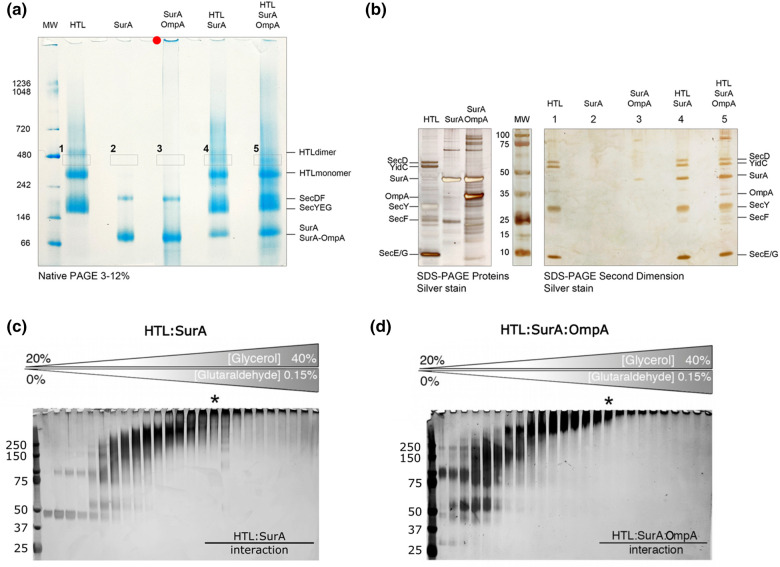
Complex formation between HTL and SurA or SurA:OmpA through NativePAGE and density gradient centrifugation. (**a**) NativePAGE analysis of HTL, SurA, SurA:OmpA alone and mixtures of HTL:SurA and HTL:SurA:OmpA. (**b**) Bands 1–5 from (**a**) were excised at the same height and analysed by SDS–PAGE by silver staining revealing the presence of protein complexes. An amount of 1 µg of HTL, SurA and SurA:OmpA were run in parallel and silver stained as markers. (**c**,**d**) Silver stained SDS–PAGE analysis of glycerol fractions following cross-linking density gradient centrifugation of the translocon with SurA chaperone alone (**c**) or engaged with OmpA substrate (**d**). The asterisks in (**c**) and (**d**) denote fractions taken for further analysis via negative stain EM.

For investigation of any possible interaction between the holo-translocon (HTL) and the periplasmic chaperone SurA, with or without a bound substrate OmpA, we used native PAGE. When run alone the HTL forms three bands, as previously seen [21], while SurA and SurA–OmpA formed two (Figure 1a). To verify their identity each band was excised and analysed by denaturing gel electrophoresis (SDS–PAGE) and high sensitivity silver staining (Supplementary Figure S1).

The lower HTL band has an apparent molecular weight (MW) of ∼150–200 kDa lower than expected (∼250 kDa), arising due to dissociation of the HTL into its constituent sub-complexes SecYEG and SecDF-YidC. Indeed, a closer inspection reveals two overlapping bands, which when applied to SDS–PAGE confirm their identity (Supplementary Figure S1, bands 3 and 4). This dissociation is as expected due to removal of stabilising lipids during native electrophoresis [21]. The next band up is the intact HTL, with an apparent MW slightly higher than expected ∼300 kDa (normal for native electrophoresis; Supplementary Figure S1, band 2). Finally, the upper band at ∼500 kDa is most likely a non-physiological aggregate (dimer) of the HTL (Supplementary Figure S1, band 1).

In respect of SurA and SurA–OmpA, both samples contained a major lower MW band of ∼80 kDa (Supplementary Figure S1, bands 6 and 8), and a minor higher MW band of roughly double the size (Supplementary Figure S1, bands 5 and 7); corresponding to monomeric and dimeric forms of the complex. Note that for SurA–OmpA the recovery of OmpA following native electrophoresis was lower than expected (compare Supplementary Figure S1, band 8 to the starting material shown in Figure 1b). This is most likely due to dissociation of the loosely associated OmpA during gel electrophoresis (see also below).

To identify a chaperone-translocon interaction, purified HTL was pre-incubated with either unoccupied SurA or with SurA–OmpA to allow for any complex association, prior to native electrophoresis. Given the identification of the bands corresponding to intact monomeric HTL (∼300 kDa) and the HTL-dimer (∼500 kDa), we looked for the appearance of an additional band between the two. A ∼400 kDa band was indeed visualised in samples containing HTL and SurA/SurA–OmpA (Figure 1a, boxes 4 and 5); its lesser prominence (compared with HTL alone) is presumably due to a low-affinity interaction. The band was again excised, along with the corresponding vacant position in the other samples, and analysed by SDS–PAGE (Figure 1b, boxes 1–5).

The results clearly show the existence of a ∼400 kDa complex containing both the HTL subunits and SurA (Figure 1a,b, regions 4 and 5). The presence of the translocon subunits in the equivalent region of the native gel from HTL alone (Figure 1a,b, region 1) is due to the HTL-dimer, which over laps with this area of the gel. Concerning the HTL-chaperone-substrate complex the OmpA is present (Figure 1b, region 5), but possibly at sub-stoichiometric levels, most likely due to dissociation from SurA during native electrophoresis (as noted above). The residual SurA recovered at the 400 kDa region in the SurA–OmpA sample (Figure 1b, region 3) is due to gel smearing, which is more evident in the SurA–OmpA sample (Supplementary Figure S1a). This particular sample was produced by refolding from 6 M urea and is prone to slight precipitation, evidenced also by the insoluble material in the native gel (Figure 1a, red dot). Taken all together so far, the results confirm that SurA interacts with the HTL, and suggest that the OmpA is only weakly associated, which is not very surprising given that its destiny lies elsewhere.

**Figure 2.**
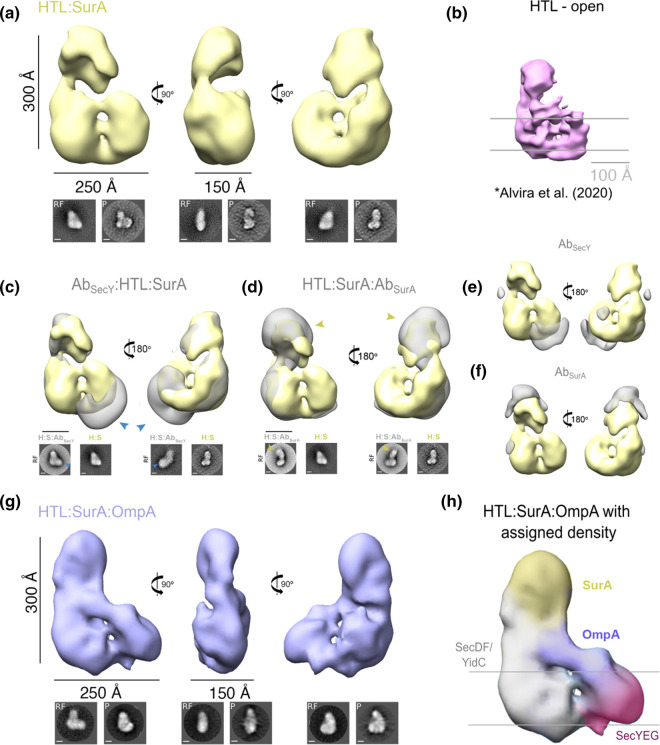
Negative-stain analysis of HTL:SurA and HTL:SurA–OmpA complexes. For a, c, d and g top panel shows views of 3D reconstructions, and bottom shows reference-free (RF) class averages and projections (P) of the final model, shown in the same orientations as the top. Scale bars are 100 Å unless stated otherwise. See Supplementary Figures S3 and S5 for image processing details. (**a**) Opposing views of the HTL:SurA complex 3D reconstruction (41.9 Å resolution). (**b**) To-scale negative stain EM density of HTL alone from [23] included for size comparison. (**c**) Opposing side views of the HTL:SurA complex (yellow) superimposed on the immuno-complex AbSecY :HTL:SurA complex (grey). The antibody (blue arrows) is shown bound to the mass assigned to SecYEG. (**d**) Opposing side views of the HTL:SurA complex (yellow) superimposed on the immuno-complex HTL:SurA:AbSurA complex (grey). The antibody (yellow arrows) is shown bound to the mass assigned to SurA. (**e**,**f**) Difference maps showing the immuno-complex density maps for AbSecY:HTL:SurA and SurA:AbSurA and AbSecY:HTL:SurA (grey) following subtraction of the HTL:SurA volume (yellow). (**g**) Orthogonal side views of the HTL:SurA–OmpA complex 3D reconstruction (37.5 Å resolution). (**h**) Assigned map of the HTL:SurA–OmpA complex with components coloured accordingly.

Density gradient centrifugation enables the isolation of complexes composed of lower-affinity interacting proteins. Additional gradient fixation, or GraFix, prevents any complex dissociation during the centrifugation through a gradient of increasing, but low, concentrations of cross-linkers [22]. We successfully applied this technique for the isolation of HTL bound to SurA and to SurA–OmpA (Figure 1c,d). The fractions were analysed by SDS–PAGE; as expected, the larger molecular weight fractions, at higher glycerol concentrations, were fully cross-linked with all the protein constituents migrating as a single band at the top of the gel. The cross-linked sample corresponding to the large complex of HTL bound to SurA (Figure 1c, asterisk) was subject to mass spectroscopy, to duly confirm the presence of SurA and translocon constituents (Supplementary Figure S2), followed by electron microscopy (EM; Figure 2a; Supplementary Figure S3). Note that not all translocon components were identified by mass spectrometry, due to their reluctance to fly. However, the identification of most of them: YidC, SecD, SecF and SecY, confirmed that the HTL (SecYEG–YidC–SecDF) is also contained in the chosen sample — consistent with the EM analysis shown below.

The low-resolution analysis by negative stain visualised a large object measuring ∼300 Å × 250 Å × 150 Å (Figure 2a,c–h), exceeding the expected dimensions of HTL alone, even in its ‘open’ state (∼200 Å × 200 Å × 150 Å) (Figure 2b) [23]. The larger size implicates the presence of SurA within the density, consistent with the mass spectrometry. However, the resolution was insufficient to localise the individual constituents of the stabilised HTL–SurA complex.

### SurA interacts with periplasmic regions of SecDF–YidC

To locate SurA within the complex with HTL, the density gradients were repeated following the addition of antibodies raised against either SecY (monoclonal) or SurA (polyclonal) (Supplementary Figure S4a,b). Similarly to the previous sample, negative stain EM samples were prepared (Supplementary Figure S4c,d); reconstructions of the cross-linked samples containing the antibodies were then overlaid with the reconstruction of HTL:SurA. Additional densities (yellow and blue arrows in Figure 2c,d highlighted by the difference maps in Figure 2e,f) identify the respective antibody binding sites, and thereby reveal the respective locations of SurA and SecY in the complex. The SecY monoclonal antibody recognises the cytosolic face of the protein–channel complex [24], at the opposite side to SurA, which is situated with the large periplasmic domains of SecDF and possibly also YidC. These periplasmic regions are also capable of forming interactions with hydrophobic OMP substrates [25–27]; whereas those in SecYEG are too small [4].

### SurA is situated on the HTL adjacent to the exit site of SecYEG

The SurA chaperone can be purified either alone or together with an OMP substrate, enabling the analysis as described above. Similarly to HTL:SurA, the high molecular weight complex containing OmpA (Figure 1d, asterisk) was also analysed by negative stain electron microscopy (Supplementary Figure S5) to obtain low-resolution structural information of the substrate engaged assembly (HTL:SurA:OmpA; Figure 2g). When comparing the structures of the occupied and vacant chaperone–HTL complex, an extra density is apparent in the former which can be assigned to the OmpA substrate (Figure 2h). The positioning of OmpA makes contact with the periplasmic surface of SecYEG, localised by the monoclonal antibody against SecY on the cytosolic side of the channel (Figure 2c,e). At this position it is roughly adjacent to the protein–channel exit site and in contact with SurA, possibly representing a late stage inner-membrane translocation intermediate.

### OmpA stabilises interactions between HTL and the SurA chaperone

**Figure 3.**
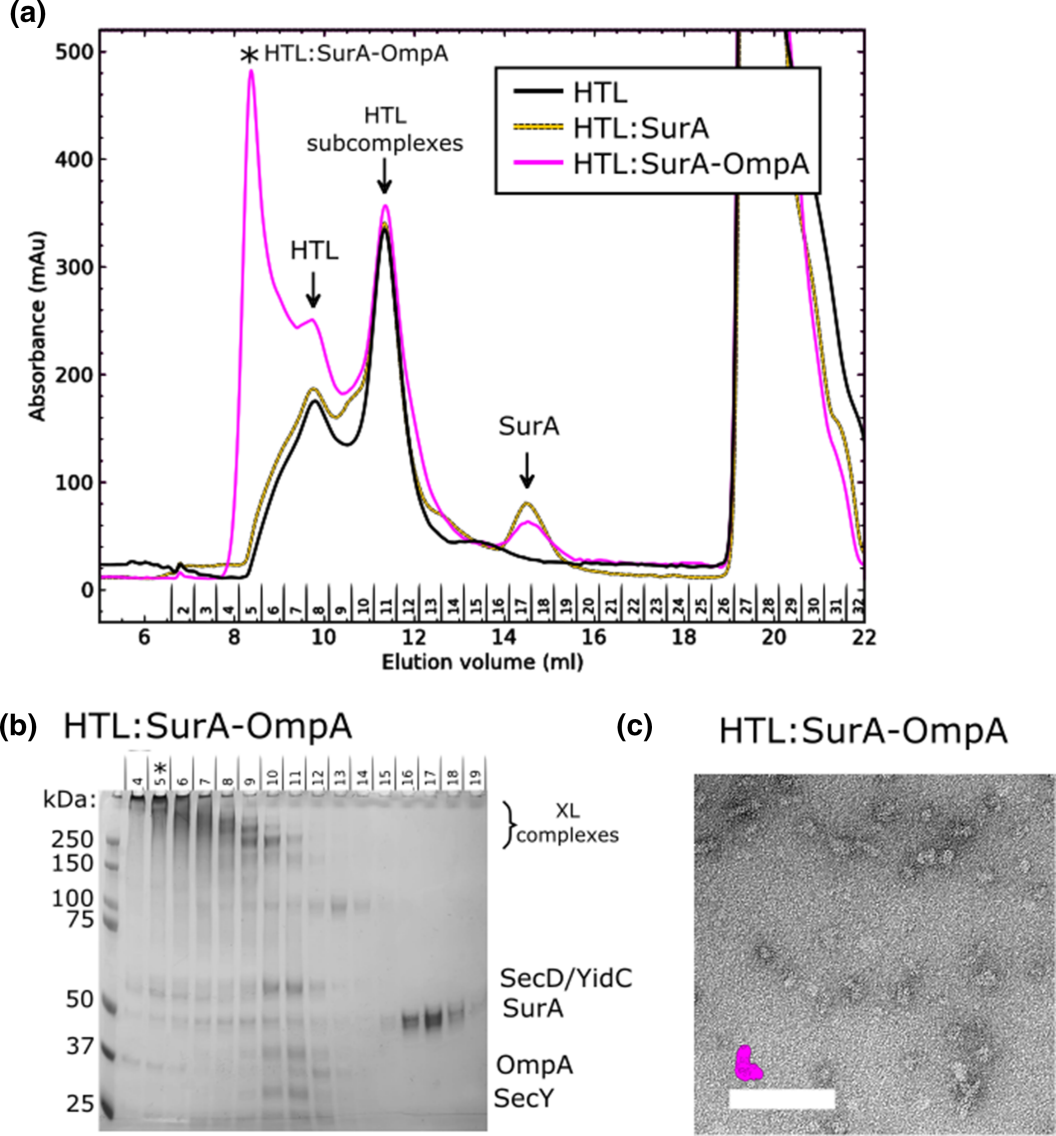
HTL:SurA–OmpA complex isolation by size-exclusion chromatography. (**a**) Size exclusion chromatograms for cross-linked samples containing purified HTL alone (black) or pre-incubated with SurA (yellow dashed) or SurA–OmpA complexes (magenta). (**b**) SDS–PAGE analysis of fractions corresponding to the chromatogram in (**a**). (**c**) Negative stain micrographs prepared from fractions highlighted in (**b**) with an asterisk. Scale bars represent 1000 Å. For indication of correct complex size, the reconstruction from the GraFix experiment for HTL:SurA–OmpA is depicted in scale in magenta.

The processing workflows of the negative stain EM data reveals that there is a large degree of flexibility between SurA and the HTL within the HTL:SurA complex (Supplementary Figure S3), which is much reduced in the presence of OmpA (Supplementary Figure S5). This suggests OmpA stabilises the chaperone-translocon interaction. To further explore this effect we employed size-exclusion chromatography, in the presence of a low concentration of an amide cross-linker (to stabilise the low-affinity interactions). This was conducted for the translocon alone, or following addition of unoccupied or substrate-engaged SurA (Figure 3a). In the presence of the OMP substrate there is a large peak at a higher apparent molecular weight, compared with the translocon alone, or empty chaperone bound complex (Figure 3a, asterisk). Subsequent, analysis of this fraction by SDS–PAGE and negative stain EM (Figure 3b,c) confirms the presence of a cross-linked complex of the similar dimensions and shape to the complexes isolated by density gradient centrifugation (Figures 1c,d and 2). The large shift of OmpA engaged chaperone–translocon substrate is indeed consistent with change in the dynamic properties of the complex, compared with the unoccupied complex. This is in keeping with an expected response to the presence of OmpA and known changes of inter-domain dynamics of SurA upon interaction with its clients [13].

### Low-resolution cryo-EM structure analysis reveals complex disorder

**Figure 4.**
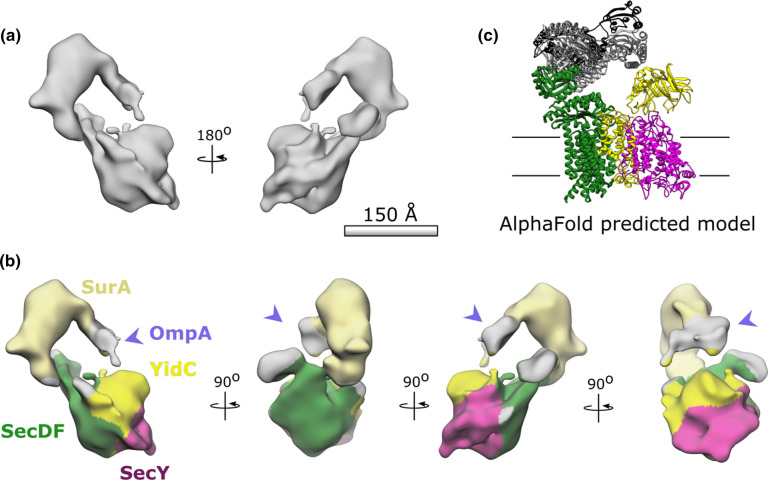
Low resolution cryo-EM of HTL:SurA:OmpA. (**a**) 22 Å resolution reconstruction processed using Relion, see Supplementary Figure S6 for image processing details. (**b**) Locations for SurA (light yellow) and SecY (magenta) are both consistent with negative stain analysis in Figure 2 and the Alphafold prediction in (**c**). Locations of SecDF (green) and YidC (yellow) have been mapped relative to SecYEG according to PDB:5MG3 [31]. The purple arrow indicates extra density likely representative of OmpA. (**c**) The top five ranked AlphaFold predictions for the interactions between SurA (different shades of grey) and SecDF (green) from *E. coli* and *Thermus thermophilus* respectively. YidC (yellow) and SecYEG (magenta) have been added according relative positions in the published HTL structure PDB:5MG3 [31].

Cryo-EM analysis of the high-molecular weight peak from the HTL:SurA:OmpA size exclusion chromatography (Figure 3a, asterisk; Supplementary Figure S6) resulted in the reconstruction shown in Figure 4a. By comparison to the negative stain EM structures (Figure 2) we can assign the locations of SurA and the constituents of the HTL. The low-resolution attainable is an unfortunate consequence of the known inherent flexibility of the HTL [23,28] and SurA (see below), required for their function. The associated OmpA is presumably also very flexible and not well defined at this resolution. The poorly resolved unfolded OmpA likely contributes to the diffuse density between the chaperone and the membrane domain (Figure 4b, arrow); this density was also evident in the negative stain analysis (Figure 2g,h).

Note the propensity of OmpA to dissociate from SurA resulting in a sub-stoichiometric HTL:SurA:OmpA complex (seen above; Figure 1b) is not the reason for the poorly resolved density assigned here. This is because: (1) the complex had not undergone native electrophoresis, a likely cause of OmpA dissociation; (2) the samples used for electron microscopy were subject to cross-linking to ‘lock-in’ weakly interaction proteins, and finally (3) the particles have undergone computational ‘purification’ whereby classes of complexes missing OmpA would have been cast aside (Supplementary Figure S6).

It is now established that SurA is highly dynamic [13–16] with several different conformations for OmpA binding [16]. With this in mind, it is unlikely that there is a single conformation for the HTL:SurA:OmpA complex, which is problematic for sample preparation and image processing (Supplementary Figure S6). Despite the low resolution achievable the negative stain and cryo-EM have been able to confirm an important interaction of a periplasmic chaperone with the Sec translocon for collection of nascent envelope proteins from the SecY-channel.

### AlphaFold predicts an interaction between SecDF and SurA

Through AlphaFold2 [29] we built a model for the most likely interaction between the HTL and the chaperone (Figure 4c) and used this to inform our prediction of the subunit organisation depicted in Figure 4b. Each of the core components of HTL was run through the structural prediction software in the presence of the SurA chaperone.

The SurA interactions with YidC and SecYEG (Supplementary Figure S7e,f) were varied but consisted mostly with cytosolic or membrane regions of the inner membrane proteins, and hence unfeasible within the context of the membrane envelope. However, all five top ranked models for interactions between SurA and SecDF showed SurA bound with the periplasmic P1-head domain of SecDF in an extended I-form conformation (Supplementary Figure S7b,d) (observed in [30]). The top ranking prediction of the SecDF:SurA interaction was then contextualised within the HTL using known subunit organisation [31] to produce the model in Figure 4c. This has striking similarities to our observed densities from both negative stain and cryo-EM. Additionally, the SurA in the five models predicted by AlphaFold presumes a variety of conformations consistent with the observed structural heterogeneity.

Importantly however, in all the models predicted by AlphaFold the interaction site between the SurA chaperone and SecDF was consistent. Each model showed an interaction between the P1 and P2 parvulin-like PPIase domains of SurA and the periplasmic P1 head domain of SecDF. This is of particular interest as the P1 domain in SecDF is thought to have polypeptide binding capabilities, and moves in response to proton transport [30].

Additionally, previous AlphaFold models for SecDF alone (entry AF-Q5SKE6-F1 on the AlphaFold Protein Structure Database) predict the inactive super-F form conformation [32]. However, in the presence of SurA AlphaFold predicts the active, extended form of SecDF. This suggests a preferred conformation of SecDF for SurA binding, which could be regulated during proton transport driven by the PMF.

## Discussion

**Figure 5.**
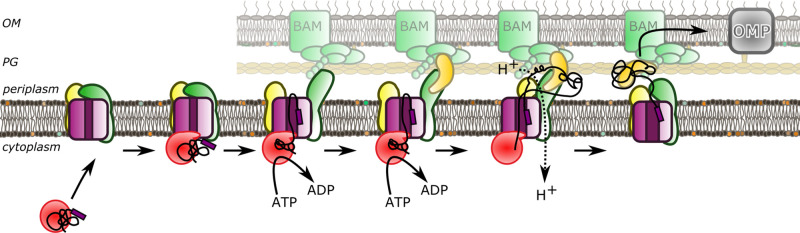
A model for OMP secretion across the bacterial envelope. OMPs are targeted to the inner membrane via an association between SecA (red), and the holo-translocon (SecY;magenta, SecDF;dark green, YidC;yellow). Translocation across the inner membrane is driven by ATP hydrolysis by SecA and the PMF (not shown). SurA (gold) interacts with the OMP during the translocation process through interactions with SecDF (green) and possibly YidC (yellow) with the OMP. OMP targeting to the outer membrane insertion machinery BAM (bright green) is assisted through interactions between the HTL and BAM and/or between SurA and BAM. Outer membrane insertion can then occur through the BAM machinery.

From the findings shown in this paper we can now present an updated model for OMP transport (Figure 5). We have established that SurA can interact with the large periplasmic domains of the HTL; presumably with those of SecDF – already known to facilitate protein secretion [33,34], and possibly also YidC. Recently, we have proposed that SecDF facilitates the onward passage of OMPs to the outer-membrane through a direct interaction with BAM [23]. We also speculated that the periplasmic chaperones SurA, Skp, PpiD and YfgM are also involved. The results described here suggest that this is likely to be true, at least in the case of the former candidate. The disposition of SurA is ideal for welcoming proteins as they emerge through SecYEG. The intrinsic dynamic properties of SurA and the periplasmic regions of SecDF, noted here and elsewhere [13–16,23,25,27,28,30,31], are presumably exploited during the passage of proteins from the Sec-machinery to BAM.

In the updated model, while pre-protein translocation is still underway — driven through SecYEG by the motor ATPase SecA and the PMF — the emerging protein immediately checks in with SurA (or perhaps another suitable chaperone), which prevents misfolding or aggregation prior to substrate release (Figure 5). Consistent with this, *surA* mutants have previously been shown to affect OMP release from the Sec-machinery [19]. Following release from the inner-membrane SurA and its client are presumably then free to move in order to facilitate OMP transfer to the connected BAM for outer-membrane insertion. Potentially, the free energy available in the PMF, driving cyclical conformational changes of the connected domains of SecDF [30], and possibly YidC, drive this process in the desired direction; i.e. a net flow of protein to the outer-membrane (Figure 5). This could be achieved via interactions of HTL periplasmic regions with the chaperone and OMP directly (shown here), in addition to those previously seen with BAM [23]. The respective domains of SecDF and YidC both contain clefts suitable for binding hydrophobic stretches of polypeptides typical of OMPs [26,30], potentially for direct interactions *en route* to the outer-membrane.

It seems likely that the associated chaperone maintains the OMPs in an unfolded aggregation free state prior to their release to BAM. Interestingly, the recently described Sec-BAM super-complex contains a central cavity, between the two membranes, large enough to accommodate small chaperones like SurA [23]. Given the known interaction of SurA with both HTL and BAM, it is possible that it could be very intimately associated within the *inner sanctum* of a trans-envelope super-complex. Alternatively, SurA could form part of the walls or gate of the periplasmic chamber. A protected environment like this would of course help prevent aggregation, proteolysis and provide an easier route through the periplasm and PG layer. In addition, the open nature of this cavity would also allow lateral escape of periplasmic resident proteins for folding, and for the release of misfolded proteins for degradation. In this respect, SurA may also help distinguish globular proteins destined for the periplasm, from β-barrelled outer-membrane proteins, and thereby regulate their lateral release and folding.

In addition to the role in outer-membrane targeting, chaperone binding of the OMP substrate during the translocation process could also help drive the translocation process itself. One of the models for SecA-dependent transport through the inner membrane suggests that the SecY channel facilitates a Brownian ratchet mechanism for biased diffusion through the channel [35,36]. Here backsliding of positively charged and bulky residues is prohibited by channel closure following retraction of the 2-helix finger of SecA from SecY. Chaperone binding to the unfolded protein substrate during translocation would curtail backward diffusion of the substrate through SecY and thereby contribute to the ratchet and promote translocation. Protein folding in the periplasmic cavity of SecY has been shown to have a similar effect [37].

Together, these findings present a novel interaction which improves our understanding of protein secretion in Gram-negative bacteria. The HTL–SurA complex is particularly important as it is unique to bacteria and therefore presents a promising target for therapeutic inhibition by antibiotic development.

## Methods

### Lipids

*Escherichia coli* polar lipid and cardiolipin (CL) were purchased from Avanti, and were prepared at 10 mg/ml in 50 mM triethanolamine, pH ∼ 7.5, 50 mM KCl.

### Cell strains

Chemically competent C43 cells were used for expression of HTL. These were cultured from home-made stocks of cells gifted by Prof. Sir John Walker (MRC, Mitochondrial biology unit, Cambridge). Expression of all soluble proteins used chemically competent home-made stocks of *E. coli* strain BL21(DE3) originally sourced commercially (NEB).

### Protein expression

#### HTL

Chemically competent C43 cells were used for expression of HTL [38]. HTL was purified as described previously [21,39,40] in TS_130_G buffer (20 mM Tris–HCl^pH ≈ 8^, 130 mM NaCl, 10% (v/v) Glycerol) for density gradient experiments and with Tris substitution for 50 mM HEPES^pH ≈ 8^ in size exclusion experiments (HS_130_G buffer: 50 mM HEPES^pH ≈ 8^, 130 mM NaCl, 10% (v/v) Glycerol) [21,39,40]. Throughout later stages of purification both buffers were supplemented with 0.02% cardiolipin (CL) as described previously [23].

#### SurA:OmpA

pET28b-*His*-*surA* and pET11a-*ompA* [41] were gifts from Prof. Sheena Radford (The Astbury Centre for Structural Molecular Biology, University of Leeds, U.K.). Expression of all SurA and SurA–OmpA proteins was carried out using chemically competent *E. coli* strain BL21(DE3). Both proteins were over-produced separately in 1 L of cultures as described previously [42]. Both were harvested by centrifugation, and resuspended in TS_130_G (20 ml 20 mM Tris pH 8.0, 130 mM NaCl, 10% (v/v) glycerol) in the presence of c*O*mplete protein inhibitor cocktail (Roche). Both were then lysed in a cell disruptor (Constant Systems Ltd.) and the resulting samples were clarified by centrifugation in an SS34 rotor (Sorvall) at 27 000×***g***, 4°C for 20 min. For OmpA, the supernatant was discarded and the pellet resuspended in 20 ml TS_130_G + 6 M urea. The OmpA pellet was diluted to 80 ml with 6 M urea and mixed with the SurA supernatant to give a final urea concentration of 4.8 M. The urea was removed by dialysing in 2 L TS_130_G for 6 h at room temperature, then dialysing overnight at 4°C in 5 L fresh TS_130_G. The sample was centrifuged again at 27 000×***g***, 4°C for 20 min and the supernatant loaded onto a 5 ml HisTrap HP column (Cytiva) equilibrated with a low salt buffer, TS_50_G (20 ml 20 mM Tris pH 8.0, 50 mM NaCl, 10% (v/v) glycerol). The column was washed with a low imidazole buffer (TS_50_G + 20 mM imidazole), and bound proteins eluted with a high imidazole buffer (TS_50_G + 300 mM imidazole). The eluents were loaded onto a HiTrap Q HP column (Cytiva) equilibrated in the low salt buffer (TS_50_G) and free SurA was found in the unbound fraction. A linear gradient of 0.05–1 M NaCl was applied over 60 ml and following SDS–PAGE analysis, fractions containing SurA–OmpA were pooled.

For size exclusion experiments, purification was repeated under identical conditions with Tris substituted with 50 mM HEPES during the purification (HS_20_G: 50 mM HEPES ^pH ≈ 8,^ 20 mM NaCl, 10% glycerol). The flow-through from HiTrap Q HP column (Cytiva) contained the SurA–OmpA under these conditions and so this was concentrated and to <500 µl and loaded to a S200 30/150 SEC column (Cytiva). Fractions from the peaks corresponding to SurA–OmpA complexes were pooled and concentrated.

### Binding assay between HTL and SurA or SurA:OmpA

HTL (3.8 µM) was incubated with purified SurA or SurA:OmpA at 1 : 3 molar ratio in TS_130_G buffer (20 mM Tris–HCl^pH ≈ 8^, 130 mM NaCl, 10% (v/v) Glycerol) supplemented with 0.01% DDM-CL for 30 min at 30°C. Individual proteins were subjected to the same procedure. Aliquots were then loaded to a native electrophoresis using NativePAGE Bis-Tris 3–12% (Invitrogen) gels run at 130 V for 1 h and additional 1.5 h at 140 V at room temperature. Protein complex bands separated in the native electrophoresis were excised, incubated for 10 min in LDS–PAGE sample buffer supplemented with 100 mM DTT, applied to the top of a SDS–PAGE gel NuPAGE 4–12%, 1 mm wells (Invitrogen) and run at 140 V for 90 min. Gel bands of the individual proteins were excised at the same height as the protein complexes as migration controls. After electrophoresis, gels were silver stained with SilverQuest^TM^, (Invitrogen) following manufacturers recommendations.

### Density gradient ultracentrifugation

Glycerol gradients for gradient fixation (GraFix) as described in [22] were prepared in a Sw60Ti ultracentrifugation tube (Beckmann) with 26 layers of 150 µl per glycerol concentration. The different concentrations were achieved by mixing a 0% glycerol gradient buffer (20 mM HEPES^pH ≈ 8^, 250 mM NaCl, 0.03% DDM-CL, with a 60% glycerol buffer (20 mM HEPES^pH ≈ 8^, 250 mM NaCl, 0.03% DDM-CL, 60% glycerol) to achieve a gradient from 20–40% glycerol without or alongside a glutaraldehyde gradient of 0–0.15%.

Different combinations of HTL alone or with either SurA or SurA:OmpA complexes were diluted in the 0% glycerol gradient buffer (20 mM HEPES, 250 mM NaCl, 0.03% DDM-CL) to a total volume of 150 µl with protein concentrations of 5 µM each. These were incubated at 30°C for 40 min followed by 5 min on ice. Protein mixtures were carefully floated on top of prepared gradients and centrifuged in a Sw60Ti rotor (Beckmann) for 16 h at 134 000×***g*** and 4°C. Each layer was manually extracted into tubes analysed by SDS–PAGE with silver staining using the fast staining protocol within SilverQuest^TM^ Silver staining kit (# LC6070 Novex).

For HTL:SurA:Ab_SurA_ and Ab_SecY_:HTL:SurA complexes, 5 µM of HTL and SurA were incubated in binding buffer at 30°C for 30 min with shaking in a total volume of 150 µl. SecY antibody was added at a dilution factor of 1 in 7.5 and incubated at 30°C for 15 min with shaking in a total volume of 150 µl.

### Size exclusion chromatography (SEC)

An amount of 10 µM of HTL and/or SurA/SurA–OmpA were mixed in 150 µl reaction volume in buffer containing 50 mM HEPES^pH ≈ 8^, 130 mM NaCl, 10% glycerol, 0.01% DDM-CL and 1 µg/ml pepstatin A. Reactions were incubated at 25°C for 30 min with shaking. Bis-(Sulfosuccinimidyl) suberate (BS3, Thermo Fisher Scientific #21580) was added to a concentration of 3.2 mM and further incubated for 40 min at 25°C with shaking. Finally 50 mM ammonium chloride was added to quench the cross-linking and this was incubated at 25°C for a further 15 min. Samples were then loaded onto a S200 10/300 column (Cytiva) equilibrated in buffer containing 50 mM HEPESpH ≈ 8, 130 mM NaCl, 0.01% DDM-CL (no glycerol). Fractions were analysed through SDS–PAGE with Coomassie staining.

### Negative stain electron microscopy

#### Sample preparation

Negative stain EM was conducted using 300 mesh copper grids coated with carbon film (agar scientific) glow discharged for 15 s. 5 µl of a suitably diluted protein sample was applied to the carbon side of the grid and incubated for ∼1 min. Grids were blotted, washed with water, re-blotted and finally stained using a 5 µl drop of 2% uranyl acetate for a further 1 min. This was blotted a final time and grids were stored and logged. All further analysis was done using either a Tecnai 12 microscope at the Living Systems Institute, University of Exeter, or the Tecnai 12 and Tecnai 20 microscopes in the Wolfson Bioimaging Suite at the University of Bristol (see below):

##### GraFix purified HTL–SurA and HTL–SurA–OmpA

Tecnai 12 (Thermo Fisher Scientific) with a One View Camera (Gatan) at a digital magnification of 59 400× and a sampling resolution of 2.1 Å per pixel (EM facility, University of Exeter).

##### SEC purified HTL–SurA–OmpA

Tecnai 20 (Thermo Fisher Scientific) with Ceta 4k × 4k CCD camera (Thermo Fisher Scientific) at a digital magnification of 50 000× and a sampling resolution of 2.02 Å per pixel (Wolfson Bioimaging facility, University of Bristol).

#### Processing

All image processing for negative stain EM was carried out in EM software framework Scipionv2.0 [43] using University of Bristol High Performance Computing specialist cluster BlueCryo. Micrographs were imported and manually picked particles provided the input toward automatic picking through the XMIPP3 package [44,45]. Particle extraction used Relion v2.1 package [46,47] and reference-free class averages grouped particles using Relion 2D classification (v3.0). The low quality classes from this output were removed and a second round of 2D classification was conducted. Similar 2D classification software XMIPP–CL2D was also carried out in parallel [48]. The outputs of these could be used to generate 3D models using EMAN v2.12 software [47,49]. Many consecutive rounds of 3D classification in Relion (v3.0) [47] were used to filter out particles and create a homogeneous set for the most detailed structure possible. This was carried out using Relion 3D-classification. 3D auto-refinement was also conducted using Relion (v3.0). Resolution estimates through Relion used 0.143 correlation coefficient criteria [50,51].

### Cryo-electron microscopy

An amount of 4 µl of marked fractions from size exclusion chromatography (asterisk, Figure 3) diluted 40 times (in 50 mM HEPESpH ≈ 8, 130 mM NaCl, 0.01% DDM-CL) were loaded on freshly prepared graphene oxide coated grids (prepared using methods described in [52]) and plunge frozen into liquid ethane using a Vitrobot Mark IV unit (Thermo Fisher Scientific) with wait time of 2 s after sample application and blotting time of 2 s. 1081 movies acquired on a TALOS Arctica (Thermo Fisher Scientific) microscope with K2 direct electron detector (Gatan) in linear mode (at GW4 facility, University of Bristol).

Patch motion correction was carried out using cryoSPARC v2.15 + 200728 4.4 within the cryoSPARC web interface [53–56]. The remainder of the processing was conducted within the Scipion v2.0 framework [43] and included use of: CTFFIND4 [57,58], Xmipp v3.0 [45,59] and Relion v3.0 [46,47,51,60].

### AlphaFold

AlphaFoldv2 [29] was carried out within Google Colab [61]. *E.coli* amino acid sequences were used for SurA (P0ABZ6) and YidC (P25714). To reduce complexity the single chain SecDF from *Thermus thermophilus* (Q5SKE6) was used. *T.thermophilus* SecDF is also used in a number of structural studies and has high levels of conservation to SecDF in *E. coli* [25,32].

### Graphical analysis

Molecular graphics and analyses was performed with UCSF Chimera v1.15, developed by the Resource for Biocomputing, Visualisation, and Informatics at the University of California, San Francisco [62]. Any map segmentation was done using the segger tool within this software [63,64]. As stated, molecular ‘docking’ was conducted by hand. The coloured map in Figure 4c used Chimera ‘Colour Zone’ following the AlphaFold model to inform the docking of HTL (PDB:5mg3) and SurA (PDB:1m5y) into the cryo-EM density.

## Data Availability

All data are available in the main text or the Supplementary Materials.

## Open Access

Open access for this article was enabled by the participation of University of Bristol in an all-inclusive *Read & Publish* agreement with Portland Press and the Biochemical Society under a transformative agreement with JISC.

## Abbreviations

BAM: β-barrel assembly machinery
CL: cardiolipin
EM: electron microscopy
HTL: holo-translocon
MW: molecular weight
OMPs: outer-membrane proteins
PG: peptidoglycan
PMF: proton-motive force
