# Supplementary material for: Interaction of the periplasmic chaperone SurA with the inner membrane protein secretion (SEC) machinery
Source: Biochem J. 2023 Feb 27;480(4):283–96. doi: 10.1042/BCJ20220480 (PMC9987972; doi:10.1042/BCJ20220480)
Supplement: Supplementary Material 1 [file BCJ-480-283-s1.pdf]

INTERACTION OF THE PERIPLASMIC CHAPERONE SURA WITH THE INNER MEMBRANE PROTEIN SECRETION (SEC) MACHINERY

Lucy Troman[1*], Sara Alvira[1], Bertram Daum[2,3], Vicki A. M. Gold[2,3] & and Ian Collinson[1†]

[1] *School of Biochemistry, University of Bristol, BS8 1TD, UK*

[2] *Living Systems Institute, University of Exeter, UK*

[3] *College of Life and Environmental Sciences, Geoffrey Pope, University of Exeter, UK*

[†], corresponding author: ian.collinson@bristol.ac.uk

[*], present address: *Birkbeck, University of London, Malet St, London WC1E 7HX, UK*

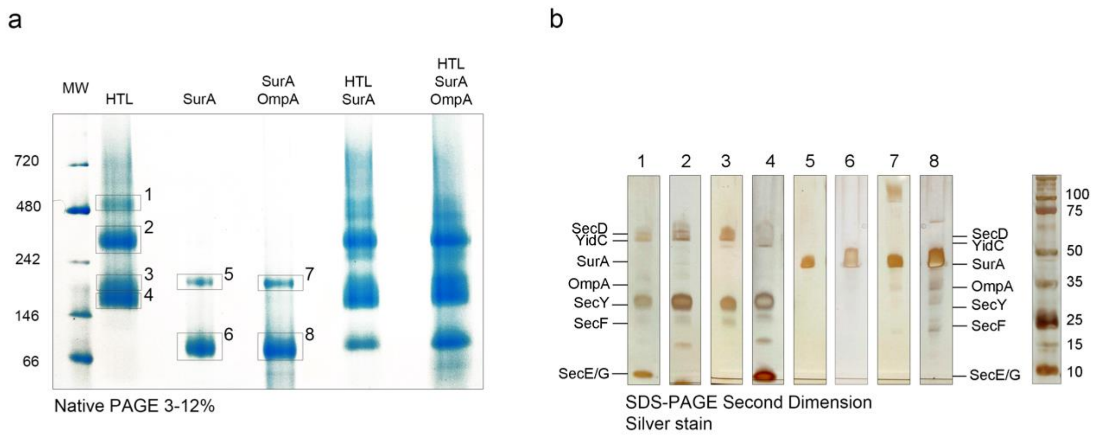

Supplementary Figure 1: **Protein composition of NativePAGE bands of HTL, SurA, SurA:OmpA and its complexes:** a) NativePAGE analysis of HTL, SurA, SurA:OmpA alone and mixtures of HTL:SurA and HTL:SurA:OmpA. The gel was previously shown in Figure 1a but it has been modified here in contrast to show clearly the HTL:SurA and HTL:SurA:OmpA bands. Boxes 1-8 denote locations of band excision. b) SDS-PAGE second dimension of bands labelled 1-8 excised from the NativePAGE gel.

Sample		Accession	Description	Score	Coverage	Prote	Peptid	# PSMs	Area
SurA:HTL detergent		B7UJN1	Protein translocase subunit SecD OS=Escherichia coli O127:H6 (strain E2348/69 / EPEC) OX=574521 GN=secD PE=3 SV=1 - [B7UJN1_ECO27]	1098.08	50.57	1	33	393	6.386E+8
		B7UMH2	Membrane protein insertase YidC OS=Escherichia coli O127:H6 (strain E2348/69 / EPEC) OX=574521 GN=yidC PE=3 SV=1 - [YIDC_ECO27]	266.32	32.66	1	13	98	1.235E+8
		B7UJN2	Protein-export membrane protein SecF OS=Escherichia coli O127:H6 (strain E2348/69 / EPEC) OX=574521 GN=secF PE=3 SV=1 - [B7UJN2_ECO27]	236.81	18.27	1	5	70	4.934E+7
		B7UIA1	Chaperone SurA OS=Escherichia coli O127:H6 (strain E2348/69 / EPEC) OX=574521 GN=surA PE=3 SV=1 - [B7UIA1_ECO27]	49.97	23.60	1	10	23	3.360E+6
		B7UK24	Protein translocase subunit SecY OS=Escherichia coli O127:H6 (strain E2348/69 / EPEC) OX=574521 GN=secY PE=3 SV=1 - [B7UK24_ECO27]	7.01	4.51	1	3	4	1.931E+6

Supplementary Figure 2: **Mass-spectrometry analysis of the HTL:SurA complex purified by density gradient centrifugation**: The fraction denoted with an asterisk in Figure 1c was taken for this analysis. This confirms the presence of SecD, YidC, SecF, SurA and SecY within the sample.

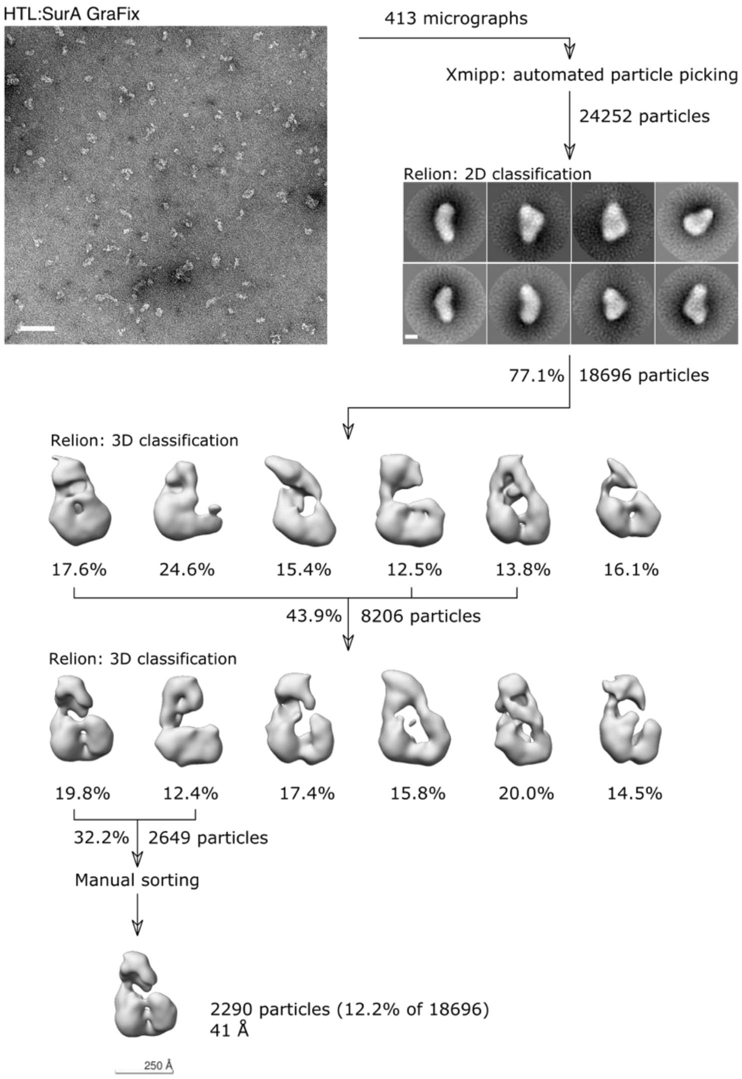

Supplementary Figure 3: **Negative-stain HTL:SurA processing pipeline:** Images of 413 micrographs yielded 24,252 particles following picking, of which a subset of 18,696 quality particles were isolated through 2D classification. Following Relion 3D classification, classes revealed a variety of conformations and particle qualities. Three of the most similar classes were chosen with 8,206 particles. A second classification followed by a manual particle sort prior to relion autorefine resulted in a low-resolution structure of 41 Å containing only 12.2% of the original particles. Scale bars for the micrograph (top left) and reference free 2D class averages (top right) represent 1000 Å and 100 Å respectively.

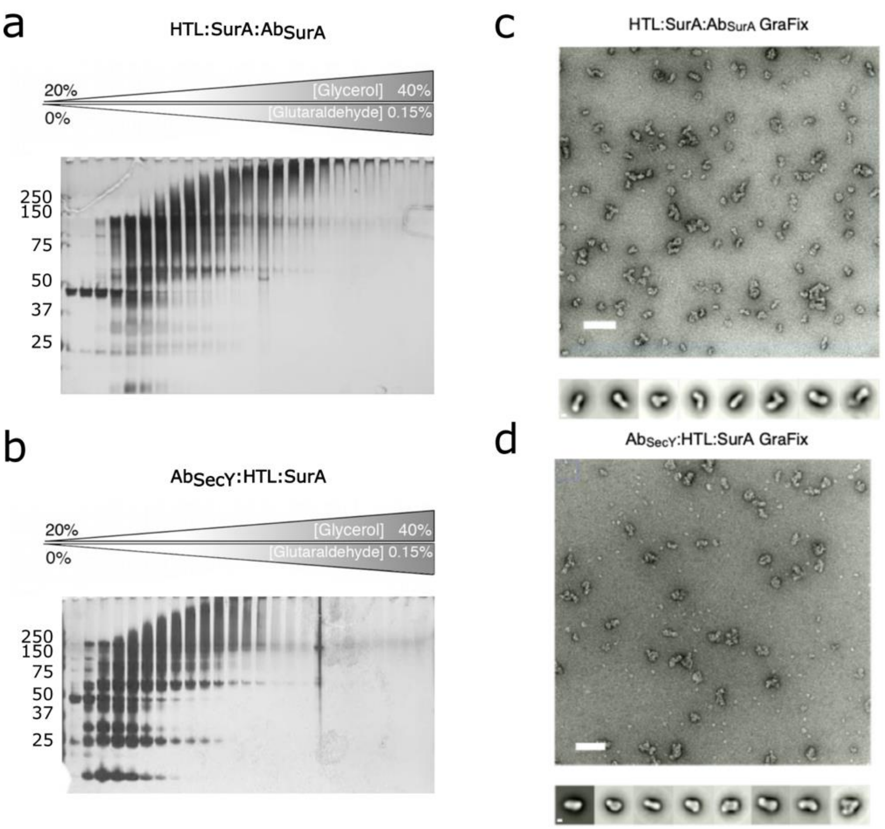

Supplementary Figure 4: **Density gradient centrifugation of the translocon and chaperones alongside SurA and SecY antibodies:** a,b) Silver stained SDS-PAGE analysis of glycerol fractions following density gradient centrifugation of the translocon with SurA chaperone repeated with polyclonal SurA antibody (a) or a monoclonal SecY antibody (b). c,d) Representative negative stain micrograph and reference free 2D class averages of a single fraction of the density gradient centrifugation with SurA antibody (a,c) or SecY antibody (b,e). Scale bars for the micrograph and reference free 2D class averages represent 1000 Å and 100 Å respectively.

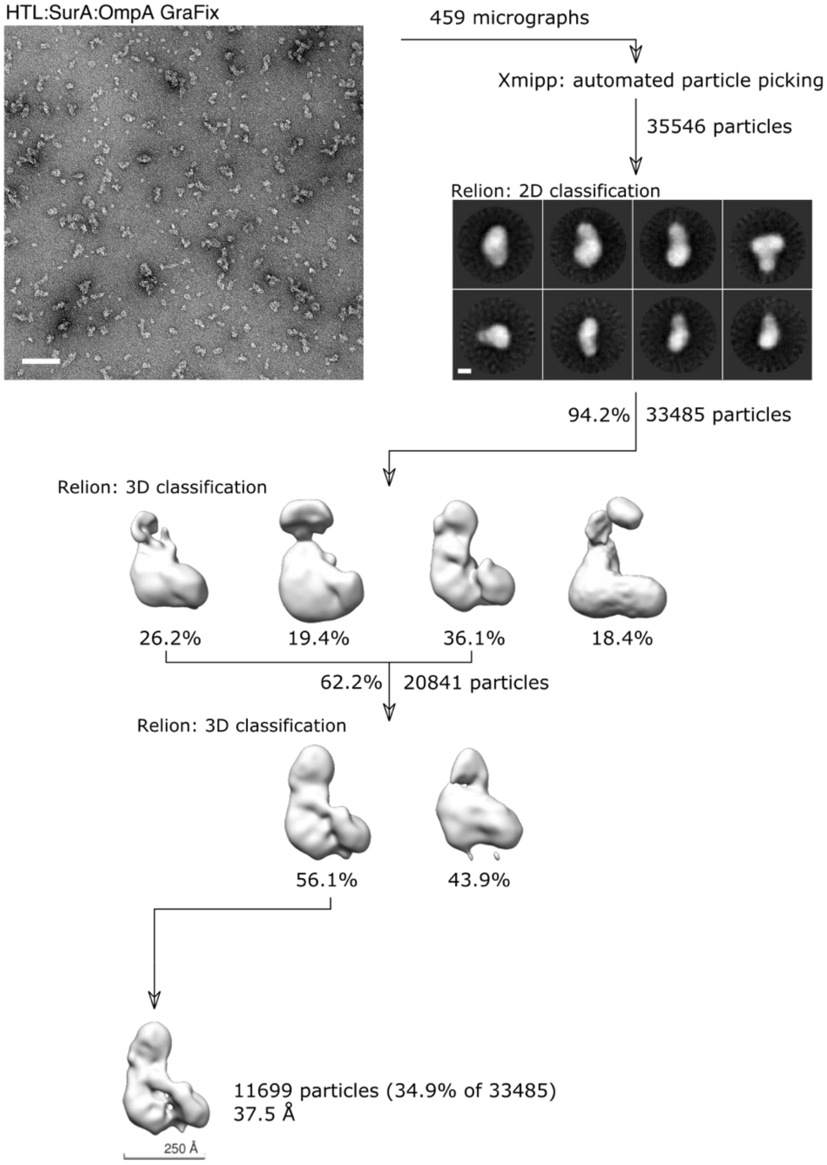

Supplementary Figure 5: **Negative-stain HTL:SurA-OmpA processing pipeline:** Images of 459 micrographs yielded 35,546 particles following picking, of which a subset of 33,485 quality particles were isolated through reference-free 2D classification. Following Relion 3D classification, classes relion autorefine resulted in a low-resolution structure of 37.5 Å containing 37.5% of the original particles. Scale bars for the micrograph (top left) and reference free 2D class averages (top right) represent 1000 Å and 100 Å respectively.

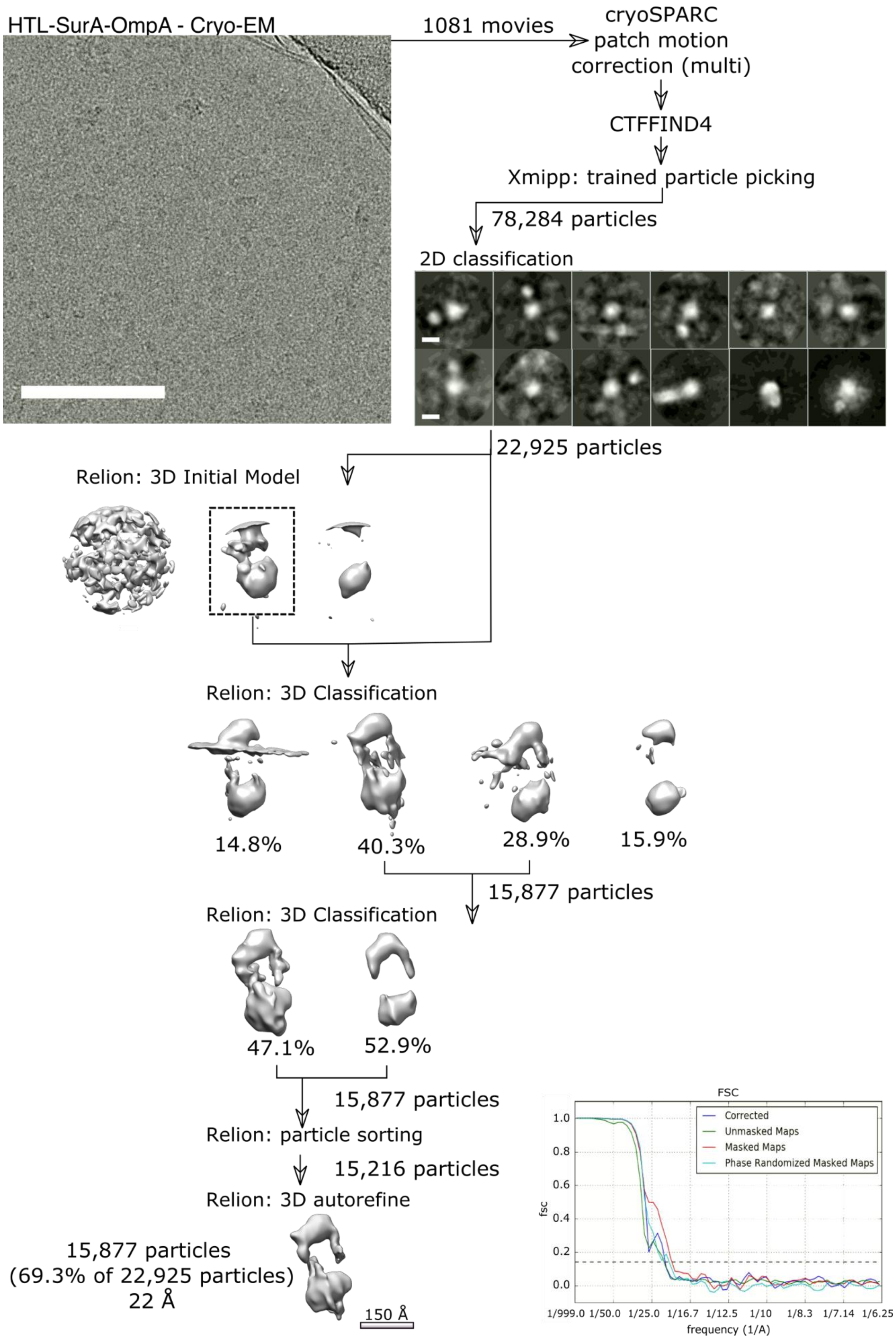

HTL-SurA-OmpA - Cryo-EM
1081 movies
cryoSPARC patch motion correction (multi)
CTFFIND4
Xmipp: trained particle picking
78,284 particles
2D classification
22,925 particles
Relion: 3D Initial Model
Relion: 3D Classification
14.8%
40.3%
28.9%
15.9%
15,877 particles
Relion: 3D Classification
47.1%
52.9%
15,877 particles
Relion: particle sorting
15,216 particles
Relion: 3D autorefine
15,877 particles
(69.3% of 22,925 particles)
22 Å
150 Å
FSC
Corrected
Unmasked Maps
Masked Maps
Phase Randomized Masked Maps
fsc
1.0
0.8
0.6
0.4
0.2
0.0
1/999.0
1/50.0
1/25.0
1/16.7
1/12.5
1/10
1/8.3
1/7.14
1/6.25
frequency (1/A)

Supplementary Figure 6: **Cryo-EM data processing of HTL:SurA:OmpA:** cryoSPARC patch motion correction aligned frames from 1081 movies, at this point micrographs were exported from processing in Scipion v2.0. Xmipp trained particle picking selected 78,284 particles, of which only 22,925 appeared the right size or had two clear densities (corresponding to SurA and HTL) for downstream processing. These were used to generate a reference-free initial model to inform two consecutive rounds of 3D classification. Following particle sorting a final run of relion 3D autorefine generated a map using 22,925 particles, or 69% of the particles selected following 2D classification. Resolution estimates through Relion gave a final resolution of 22 Å using the 0.143 correlation coefficient criteria, the plot of the Fourier shell correlation is shown in the bottom right. Scale bars for the micrograph (top left) and reference free 2D class averages (top right) represent 1000 Å and 100 Å respectively.

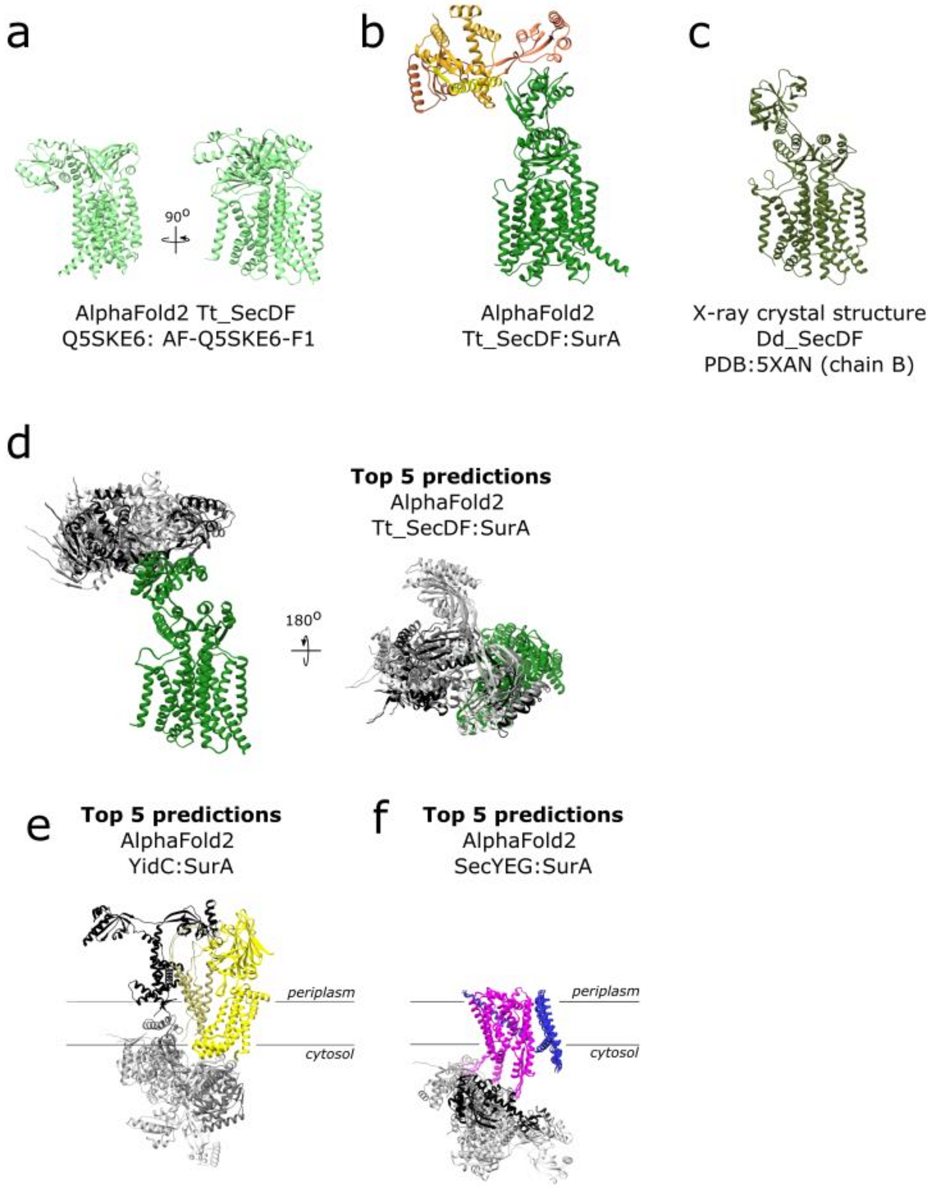

Supplementary Figure 7: **AlphaFold predictions for the HTL chaperone interaction:** a) AlphaFold predicted structure for SecDF from *Thermus thermophilus* (result number AF-Q5SKE6-F1 on AlphaFold protein structure database). b) The top ranking result for AlphaFold2 run with SecDF from *Thermus thermophilus* and SurA from *E.coli.* c) X-ray crystal structure of SecDF in I- form conformation from *Deinococcus radiodurans R1* PDB:5XANb [30]. d) The top 5 predictions for AlphaFold2 run with SecDF (green) from *Thermus thermophilus* and SurA from *E.coli* (in different shades of grey) from the side and viewed from the top. e) The top 5 predictions for AlphaFold2 run with YidC (yellow) and SurA (in different shades of grey) from *E.coli.* f) The

top 5 predictions for AlphaFold2 run with SecY (magenta), SecE (blue) and SurA (in different shades of grey) from *E.coli*.
